# Association of maternal exposures with adiposity at age 4/5 years in white British and Pakistani children: findings from the Born in Bradford study

**DOI:** 10.1007/s00125-017-4457-2

**Published:** 2017-10-24

**Authors:** Jane West, Gillian Santorelli, Peter H. Whincup, Lesley Smith, Naveed A. Sattar, Noel Cameron, Diane Farrar, Paul Collings, John Wright, Debbie A. Lawlor

**Affiliations:** 1 Bradford Institute for Health Research, Temple Bank House, Bradford Royal Infirmary, Duckworth Lane, Bradford, BD9 6RJ UK; 2 MRC Integrated Epidemiology Unit at the University of Bristol, Rm OS11, Oakfield House, Oakfield Grove, Bristol, BS8 2BN UK; 3 Population Health Science, Bristol Medical School, University of Bristol, Bristol, UK; 4 Population Health Research Institute, St George’s, University of London, London, UK; 5 Faculty of Medicine & Health, University of Leeds, Leeds, UK; 6 Institute of Cardiovascular & Medical Sciences, University of Glasgow, Glasgow, UK; 7 School of Sport, Exercise and Health Sciences, Loughborough University, Loughborough, UK

**Keywords:** Adiposity, Children, Pregnancy glycaemia, South Asian

## Abstract

**Aims/hypothesis:**

There is evidence that, from birth, South Asians are fatter, for a given body mass, than Europeans. The role of developmental overnutrition related to maternal adiposity and circulating glucose in these ethnic differences is unclear. Our aim was to compare associations of maternal gestational adiposity and glucose with adiposity at age 4/5 years in white British and Pakistani children.

**Methods:**

Born in Bradford is a prospective study of children born between 2007 and 2010 in Bradford, UK. Mothers completed an OGTT at 27–28 weeks of gestation. We examined associations between maternal gestational BMI, fasting glucose, post-load glucose and diabetes (GDM) and offspring height, weight, BMI and subscapular skinfold (SSF) and triceps skinfold (TSF) thickness at age 4/5 years, using data from 6060 mother–offspring pairs (2717 [44.8%] white British and 3343 [55.2%] Pakistani).

**Results:**

Pakistani mothers had lower BMI and higher fasting and post-load glucose and were twice as likely to have GDM (defined using modified WHO criteria) than white British women (15.8% vs 6.9%). Pakistani children were taller and had lower BMI than white British children; they had similar SSF and lower TSF. Maternal BMI was positively associated with the adiposity of offspring in both ethnic groups, with some evidence of stronger associations in Pakistani mother–offspring pairs. For example, the difference in adjusted mean BMI per 1 kg/m^2^ greater maternal BMI was 0.07 kg/m^2^ (95% CI 0.05, 0.08) and 0.10 kg/m^2^ (95% CI 0.09. 0.11) in white British and Pakistani children, respectively, with equivalent results for SSF being 0.07 mm (95% CI 0.05, 0.08) and 0.09 mm (95% CI 0.08. 0.11) (*p* for ethnic difference < 0.03 for both). There was no strong evidence of association of fasting and post-load glucose, or GDM, with outcomes in either group.

**Conclusions/interpretation:**

At age 4/5 years, Pakistani children are taller and lighter than white British children. While maternal BMI is positively associated with offspring adiposity, gestational glycaemia is not clearly related to offspring adiposity in either ethnic group.

**Electronic supplementary material:**

The online version of this article (10.1007/s00125-017-4457-2) contains peer-reviewed but unedited supplementary material, which is available to authorised users.

## Introduction

Increasing evidence suggests that greater maternal adiposity, circulating glucose and gestational diabetes (GDM) cause, via intrauterine mechanisms, greater offspring birthweight and ponderal index at birth [1–3]. Even among non-diabetic mothers there is a linear association between fasting and post-challenge glucose concentrations during pregnancy and higher birthweight and greater birth adiposity [4, 5]. Beyond birth, there is evidence that exposure to GDM in-utero results in greater offspring adiposity and overweight/obesity in infancy and early adulthood (to date there have been no reports for older ages) [1, 6] but few studies have reported associations with fasting and post-load glucose following universal oral glucose tolerance testing, as opposed to studies in which the OGTT was confined to at-risk women. The latter is important, as studies in which universal risk factor screening followed by OGTT in only those with risk factors might result in bias due to preferential diagnosis testing in obese women [1, 7, 8].

South Asian adults have a characteristic phenotype of proportionately greater adiposity, increased insulin resistance and higher rates of diabetes and cardiovascular disease compared with white European adults [9–12]. Recent evidence suggests that these differences are present in children at age 9–10 years [13] and at birth [14–18]. We have previously shown (in the same cohort as this study) that despite having notably lower birthweight, infants of Pakistani-origin mothers had higher total fat mass (as indicated by cord-blood leptin levels) and similar skinfolds in comparison with infants of white British women [18, 19]. Subsequently, we showed that greater circulating fasting and post-load glucose in Pakistani women at least partly explained the ethnic difference in birth fatness as measured by cord-blood leptin [20]. There is inconsistent evidence regarding the role of maternal fasting and post-load gestational glucose in ethnic differences in offspring adiposity beyond birth or infancy [8, 21] and, to our knowledge, no studies have explored whether maternal BMI and glycaemia traits differ in their associations with offspring measures of growth and adiposity between white European and South Asian mother–offspring pairs.

The aims of this study were as follows: (1) to determine the magnitude and direction of any differences in weight, height, BMI and triceps and subscapular skinfolds between white British and UK-born Pakistani-origin children at age 4/5 years and (2) to compare associations of maternal early pregnancy BMI, fasting and post-load glucose and GDM with offspring weight, height, BMI and triceps and subscapular skinfolds at age 4/5 years between white British and Pakistani mother–offspring pairs participating in the Born in Bradford (BiB) cohort study, all resident in the same UK city and receiving the same antenatal care.

## Methods

### Participants

The BiB cohort is a prospective pregnancy and birth cohort based in Bradford, the sixth largest city in the UK. The study recruited women during pregnancy and has followed them, their partners and their children through childhood. To be eligible, women had to attend an antenatal booking clinic between March 2007 and December 2010 and be booked to give birth in Bradford. Full details of the study methodology have been reported previously [22]. Most of the women were recruited at their OGTT appointment; all women booked for delivery in Bradford are offered a 75 g OGTT at around 26–28 weeks’ gestation. Women who attended this appointment and gave informed consent completed an interviewer-administered questionnaire and had their height and weight measured. Interviews were conducted in English as well as a range of South Asian languages (including Mirpuri, Bengali, Punjabi). A small number of women were recruited at later antenatal clinics or at the birth of their child.

**Fig. 1:**
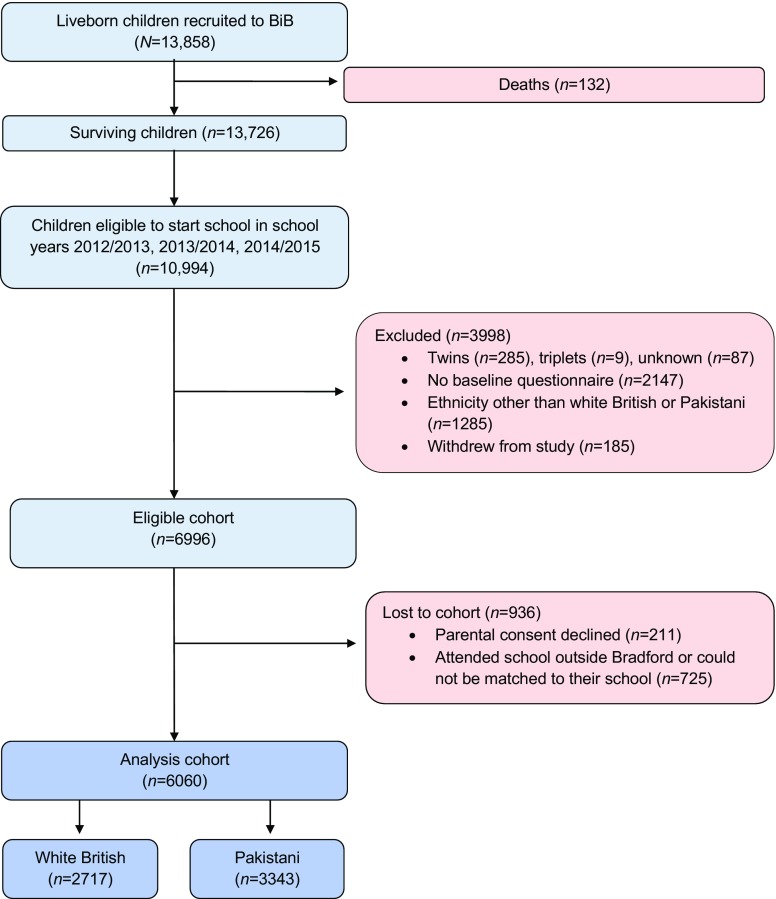
Study flow chart. ‘Unknown’ indicates no data available for these cases

Figure 1 shows the flow of participants through the study to those included in the analyses presented here. A total of 12,453 women who gave birth to 13,858 children were recruited to the BiB cohort study. Of these children, 10,994 were eligible to start school in the academic years 2012/2013, 2013/2014 and 2014/2015. For this study, we assessed the weight, height and skinfold thicknesses of BiB children in these school years, with measurements undertaken by school nurses. Mother–offspring pairs of ethnic groups other than white British and Pakistani were excluded because they included too few participants within each group for meaningful analyses. We also excluded children from twin and triplet pregnancies, those with no baseline questionnaire (because recruitment took place later than the antenatal OGTT) and those who had withdrawn from the study (Fig. 1). This left an eligible cohort of 6996. Of these, 211 (3%) of parents declined consent for their child’s measurements (with similar proportions in each ethnic group: white British 2.6% and Pakistani 3.4%). In addition, 725 (10.4% of all eligible participants, 15.1% of white British participants and 6.4% of Pakistani participants) children were excluded because they could not be matched to their school or did not attend a Bradford school, as we were unable to work with schools outside the Bradford local authority area. Thus, our final analysis sample included 6060 mother–offspring pairs (2717 white British and 3343 Pakistani).

All children in the UK at this age have height and weight measured as part of the UK Government’s National Child Measurement Programme (NCMP). Parental opt-out consent and child assent is used for those measurements, and we adopted the same approach for skinfold measurements. Ethics approval for the study was granted by Bradford National Health Service Research Ethics Committee (ref 06/Q1202/48).

### Patient involvement

BiB is a general population cohort rather than a study of patients but includes a Participant Advisory Group made up of participants in the study. This group provides comments and feedback on the development of the study and its research and these have contributed to and influenced the work presented here.

### Assessment of ethnicity

Ethnicity was self-reported by the mother at her recruitment questionnaire interview and based on UK Office of National Statistics guidance, details of which have been previously reported [20].

### Maternal pregnancy measurements

Women were weighed and their height measured (unshod and in light clothing) at recruitment (26–28 weeks’ gestation) using digital scales (Seca, Birmingham, UK) and a Leicester Height Measure (Seca). Weight at first antenatal clinic assessment, when women were median 12 weeks’ gestation (interquartile range 11–14), was abstracted from the antenatal records and this weight, together with height measured at recruitment, was used to calculate the woman’s early pregnancy BMI. All women booked for delivery in Bradford are offered a 75 g OGTT comprising fasting and 2 h post-load samples, at around 26–28 weeks’ gestation. Plasma glucose levels were assayed immediately after sampling at the biochemistry department of Bradford Royal Infirmary using the glucose oxidase method on Siemens Advia 2400 chemistry autoanalysers and Siemens Advia Centaur assay (Camberley, Surrey, UK). GDM was defined according to modified WHO criteria [23] operating at the time these women were pregnant as either fasting glucose ≥ 6.1 mmol/l or 2 h glucose ≥ 7.8 mmol/l. Women meeting the criteria for GDM were referred for specialist joint antenatal/diabetes care within the same maternity unit.

### Offspring measurements

As part of the NCMP, height was measured by trained school nurses using the Leicester Height Measure (Seca) and weight using Seca digital scales, with children unshod and in light clothing. Where NCMP height and/or weight were missing, height and weight measurements were extracted from primary care datasets, taking those that were recorded closest in time to the skinfold thickness measurements. Subscapular skinfold (SSF) and triceps skinfold (TSF) measurements were undertaken by the school nurses following training and reliability checks [24]. Holtain Tanner/Whitehouse Calipers (Holtain, Crymych, UK) were used and all measurements were taken on the left side of the body with the child seated and their left arm only removed from clothing. All skinfold measurements were conducted within 16 weeks of the height and weight measures (mean ± SD time difference 13.5 ± 8.0 weeks). High levels of between and within school nurse reliability were found for all skinfold measurements [24].

### Other variables

Information on maternal age, smoking, maternal education, whether in receipt of means tested benefits and housing tenure was obtained at the recruitment interview. Information on parity, gestational age and sex of the infant were abstracted from medical records (full details of assessments of these variables can be found in electronic supplementary material [ESM] Table 1).

### Statistical analyses

All analyses were performed using Stata/SE software (Stata/SE 13 for Windows; StataCorp, College Station, TX, USA).

#### Aim 1

Univariable linear regression was used to explore ethnic differences in offspring height, weight, BMI and SSF and TSF thickness. Since we do not believe that characteristics which plausibly influence offspring size and adiposity (our outcomes), such as child’s age and sex, and parental socioeconomic position, diet, physical activity and smoking can influence their ethnicity (our exposure), we considered that unadjusted differences were not confounded.

#### Aim 2

Multivariable linear regression was used to examine the associations of maternal BMI, fasting and post-load glucose and GDM with offspring height, weight, BMI and SSF and TSF thickness at age 4/5 years within each ethnic group. Differences in the magnitude or direction of associations were explored by looking at the ethnic specific point estimates and including an interaction term between ethnicity and the exposures for each of these associations. For each multivariable regression model, potential confounders were decided a priori based on existing published literature and our previous BiB analyses [18, 25]. In model 1 we adjusted for offspring sex and age (in months) at child measurement. We then additionally adjusted for potential confounding by maternal age, parity, smoking, maternal BMI (for exposures of maternal fasting and post-load glucose and GDM), maternal height (when the outcome was offspring height), maternal education, housing tenure and whether anyone in the household was in receipt of means tested benefits (model 2).

#### Additional analyses

We repeated analyses of fasting and post-load glucose with offspring BMI and SSF and TSF thickness in each ethnic group having removed those with diagnosed GDM (*n* = 187 white British mothers and 529 Pakistani mothers) to test whether treatment of GDM might have influenced our results.

In some studies of ethnic differences in size and adiposity, non-BMI measurements, such as skinfolds are adjusted for BMI to determine whether one ethnic group is more adipose than another for a given BMI. Despite the logic behind such adjusted results, their interpretation is difficult because both ethnicity and SSF thickness will influence BMI and hence adjusting for this can produce spurious (non-causal) associations between ethnicity and the skinfold measures (a source of bias known as collider bias) [26]. Therefore, we have explored ethnic differences in offspring skinfolds and the associations of maternal exposures with these outcomes in each ethnic group without adjustment for offspring BMI in our main analyses; rather, we undertook additional analyses in which we did adjust for offspring BMI (model 3).

Given previous studies have found some evidence for differences in the associations of maternal gestational glycaemia and offspring adiposity between women and men [21], in additional analyses we explored associations within four subgroups: white British male, white British female, Pakistani male, Pakistani female. We examined evidence for sex differences separately within each ethnic group using a test for statistical interaction.

One study of Asian (mixed South Asian and East Asian) mother–offspring pairs resident in Singapore, found an interaction between maternal BMI and gestational glucose in their relation to offspring size [27]. Although we have not seen evidence of replication of those findings in any published studies, in additional analyses we examined whether (within each ethnic group) the association of maternal fasting glucose differed between those with early pregnancy BMI ≥ 25 kg/m^2^ and those with BMI < 25 kg/m^2^.

#### Assessing the impact of missing data

To increase statistical power and reduce bias that might occur if participants were excluded because of any missing data, we used multivariate multiple imputation with chained equations to impute missing values for any variable with some missing data. We included all variables that were used in any analyses in the multivariate multiple imputation prediction models and these were undertaken with ten switching cycles and we generated 20 imputation databases. Distributions of variables from the combined multivariable imputed datasets were similar to those of the observed data (ESM Table 1). The multivariate imputation analyses included 6060 children (Fig. 1). We also undertook analyses only including those who had complete data on all variables included (*n* = 3668).

## Results

### Ethnic differences in maternal exposures and child outcome measures

Table 1 shows the distributions of maternal and offspring characteristics for the whole cohort and separately in white British and Pakistani participants. On average, early pregnancy BMI was greater and fasting and post-load glucose lower in white British mothers than in Pakistani-origin mothers. Twice as many Pakistani mothers had GDM when compared with white British women (15.8% vs 6.9%). Boys were taller and heavier than girls in both ethnic groups, which resulted in similar mean BMI in each sex. In both ethnic groups, skinfolds (both triceps and subscapular) were larger in girls than in boys.

**Table 1:** Distributions of maternal and offspring characteristics at age 4/5 years by ethnicity and sex

Characteristic	All	White British	Pakistani origin	*p* value^a^
Maternal BMI, kg/m^2^	26.10 ± 5.76	26.84 ± 6.09	25.50 ± 5.40	< 0.001
*n*	5834	2602	3232	
Maternal glucose, mmol/l				
Fasting	4.52 ± 0.55	4.40 ± 0.42	4.61 ± 0.62	< 0.001
*n*	5836	2615	3221	
Post-load	5.67 ± 1.53	5.43 ± 1.29	5.87 ± 1.67	< 0.001
*n*	5836	2615	3221	< 0.001
Maternal GDM	716 (11.8)	187 (6.9)	529 (15.8)	< 0.001
*n*	6060	2717	3343	
Child height, cm				
Boys	108.85 ± 4.98	108.44 ± 4.84	109.21 ± 5.07	< 0.001
*n*	2556	1179	1377	
Girls	108.03 ± 4.91	107.76 ± 4.83	108.25 ± 4.97	0.012
*n*	2586	1164	1422	
Child weight, kg				
Boys	19.11 ± 2.96	19.16 ± 2.54	19.07 ± 3.28	0.422
*n*	2578	1184	1394	
Girls	18.86 ± 3.05	18.03 ± 2.85	18.72 ± 3.21	0.009
*n*	2604	1171	1433	
Child BMI, kg/m^2^				
Boys	16.07 ± 1.63	16.25 ± 1.36	15.91 ± 1.82	< 0.001
*n*	2556	1179	1377	
Girls	16.08 ± 1.75	16.33 ± 1.62	15.88 ± 1.82	< 0.001
*n*	2586	1164	1422	
Child SSF, mm				
Boys	5.82 ± 1.84	5.82 ± 1.61	5.82 ± 2.04	0.979
*n*	2224	1078	1146	
Girls	6.78 ± 2.42	6.84 ± 2.13	6.74 ± 2.63	0.340
*n*	2394	1049	1345	
Child TSF, mm				
Boys	9.58 ± 3.05	10.06 ± 2.84	9.12 ± 3.18	< 0.001
*n*	2248	1084	1164	
Girls	10.64 ± 3.40	11.41 ± 3.14	10.03 ± 3.47	< 0.001
*n*	2422	1061	1361	

Data are means ± SD or *n* (%)

^a^Difference between white British and Pakistani

**Fig. 2:**
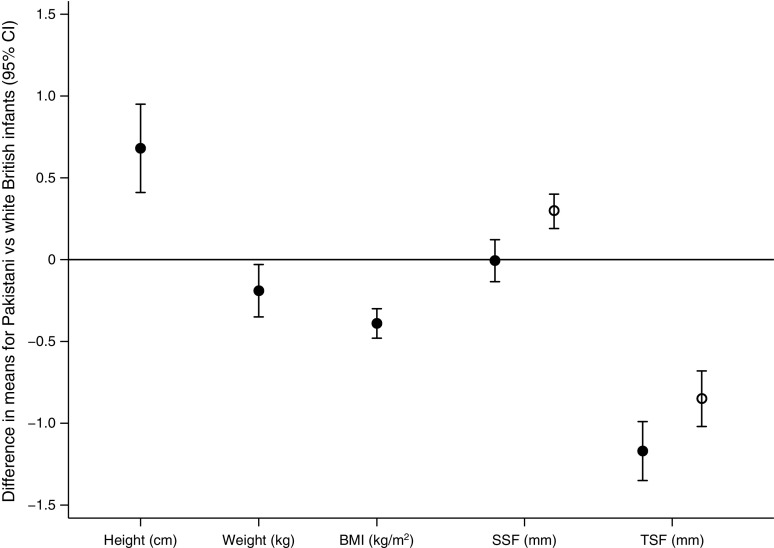
Differences in mean offspring size and adiposity between Pakistani and white British infants. Black circles, unadjusted differences in means comparing Pakistani with white British (with positive differences suggesting higher levels in Pakistani children on average); white circles, additional adjustment for birthweight. 95% CIs are indicated by capped vertical lines. Analyses are based on the multiple imputed datasets

Pakistani children were taller and had lower BMI than white British children (Fig. 2). They had similar SSF thickness and lower TSF thickness in our main (unadjusted) analyses. With additional adjustment for offspring BMI, the difference in mean SSF thickness became positive and the difference in mean TSF thickness reduced slightly (Fig. 2).

### Associations of maternal BMI and glycaemia traits with offspring size and adiposity in white British and Pakistani mother–offspring pairs

**Fig. 3:**
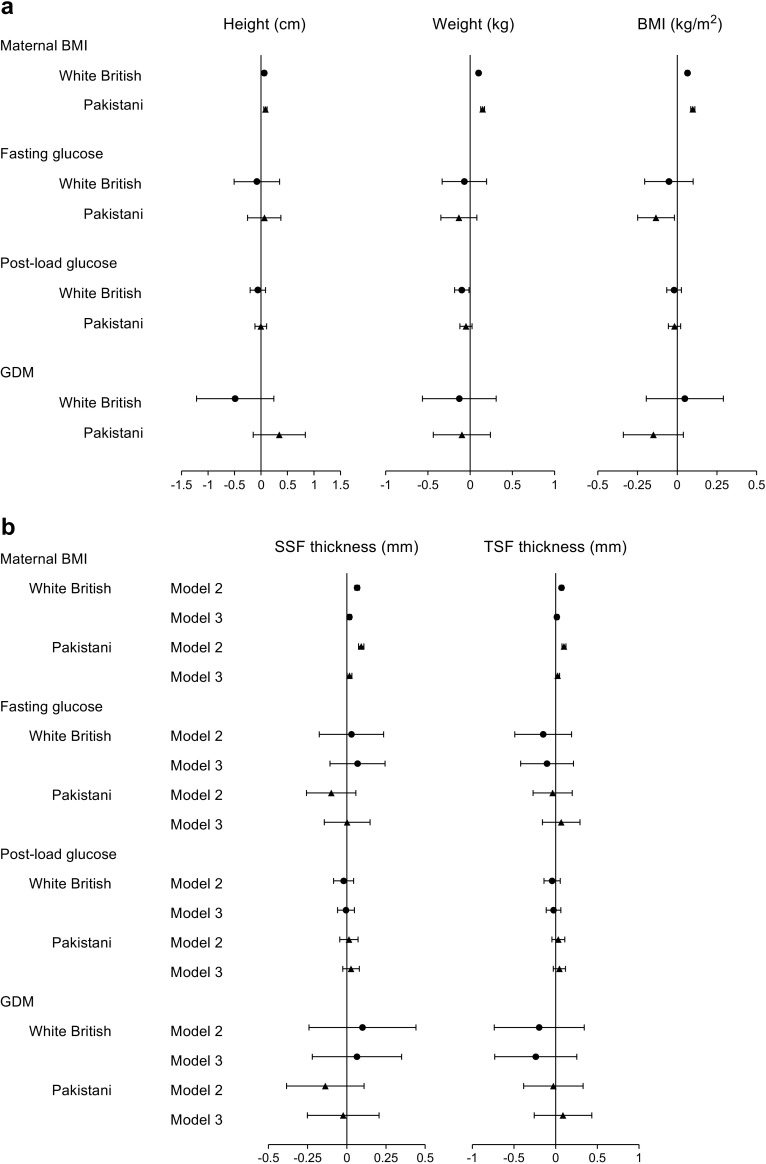
Adjusted associations of maternal pregnancy exposures with offspring variables at age 4/5 years. Data show the difference in means per unit increase in maternal BMI, fasting glucose and post-load glucose, comparing offspring whose mothers (circles, white British; triangles, Pakistani) had GDM with those who did not. 95% CIs are indicated by capped horizontal lines. Analyses were based on the multiple imputed datasets. (**a**) Adjusted associations of maternal pregnancy exposures with offspring height (cm), weight (kg) and BMI at age 4/5 years. Results are adjusted for child sex, age at child measurement, maternal age, parity, BMI (for maternal fasting and post-load glucose and GDM), maternal height (when the outcome was offspring height) and education, housing tenure, whether anyone in the household was in receipt of means tested benefits, and smoking (model 2). (**b**) Association of maternal pregnancy exposures with offspring SSF (mm) and TSF (mm) thickness at age 4/5 years. Results are adjusted for sex, age at child measurement, maternal age, parity, BMI (for maternal fasting and post-load glucose and GDM) and education, housing tenure, whether anyone in the household was in receipt of means tested benefits, and smoking (model 2) with further adjustment for offspring BMI (model 3)

Maternal BMI was positively associated with offspring height, weight, BMI and SSF and TSF thickness in both ethnic groups (ESM Table 2 shows offspring age- and sex-adjusted results [model 1]), with additional adjustment for potential confounders not notably altering these results (Fig. 3a,b and Table 2). The magnitudes of these positive associations were stronger in Pakistani than in white British mother–offspring pairs, with statistical evidence for a difference with weight, BMI and SSF. Maternal gestational fasting glucose, post-load glucose and GDM were generally inversely associated with size and adiposity outcomes in offspring but most associations were consistent with the null hypothesis in the fully adjusted models; (null) associations did not differ between the two ethnic groups (Fig. 3a,b and Table 2).

**Table 2:** Adjusted associations of maternal pregnancy exposures with offspring height, weight and adiposity

Measure	Maternal BMI, difference in means per 1 kg/m^2^ (95% CI)			Maternal fasting glucose, difference in means per 1 mmol/l (95% CI)			Maternal post-load glucose, difference in means per 1 mmol/l (95% CI)			GDM, cases vs non-cases (95% CI)		
	White British *n* = 2717	Pakistani *n* = 3343	*p* value^a^	White British *n* = 2717	Pakistani *n* = 3343	*p* value^a^	White British *n* = 2717	Pakistani *n* = 3343	*p* value^a^	White British *n* = 2717	Pakistani *n* = 3343	*p* value^a^
Height, cm	0.063 (0.032, 0.093)	0.084 (0.052, 0.116)	0.415	−0.078 (−0.506, 0.350)	0.065 (−0.245, 0.376)	0.564	−0.059 (−0.204, −0.085)	−0.003 (−0.113, 0.106)	0.577	−0.489 (−1.217, 0.241)	0.345 (−0.147, 0.838)	0.048
Weight, kg	0.101 (0.082, 0.119)	0.149 (0.126, 0.172)	0.003	−0.068 (−0.331, 0.196)	−0.132 (−0.345, 0.081)	0.719	−0.098 (−0.183, −0.013)	−0.048 (−0.120, 0.024)	0.200	−0.127 (−0.563, 0.309)	−0.096 (−0.434, 0.242)	0.504
BMI, kg/m^2^	0.065 (0.054, 0.075)	0.098 (0.085, 0.111)	<0.001	−0.052 (−0.205, 0.100)	−0.134 (−0.249, −0.018)	0.719	−0.020 (−0.066, 0.027)	−0.017 (−0.056, 0.022)	0.372	0.048 (−0.195, 0.290)	−0.150 (−0.339, 0.039)	0.707
SSF, mm	0.066 (0.052, 0.080)	0.092 (0.075, 0.109)	0.027	0.030 (−0.176, 0.235)	−0.100 (−0.257, 0.058)	0.896	−0.020 (−0.084, 0.044)	0.014 (−0.045, 0.072)	0.196	0.101 (−0.241, 0.442)	−0.137 (−0.384, 0.110)	0.637
TSF, mm	0.072 (0.051, 0.093)	0.100 (0.076, 0.123)	0.082	−0.149 (−0.490, 0.192)	−0.036 (−0.271, 0.200)	0.308	−0.042 (−0.139, 0.055)	0.032 (−0.045, 0.110)	0.135	−0.197 (−0.736, 0.343)	−0.027 (−0.384, 0.330)	0.359

All results are adjusted for offspring sex and age at measurement and maternal age, parity, BMI (for maternal fasting and post-load glucose and GDM), maternal height (when the outcome was offspring height), education, housing tenure, whether anyone in the household was in receipt of means tested benefits, and smoking (model 2). Analyses were based on the multiple imputed datasets

^a^ *p* value is for ethnic interaction, testing the null hypothesis that there is no difference in associations of maternal characteristics with offspring outcomes between white British and Pakistani

### Additional analyses

Associations were similar in female and male offspring in each ethnic group (ESM Table 3). With both ethnic groups combined (*n* = 6060), there were consistent positive associations of maternal BMI with offspring height, weight, BMI and SSF and TSF thickness and weak inverse associations, with wide CIs including the null value for associations of maternal glycaemia traits with all outcomes (ESM Table 4). Results using complete case analyses were similar, but with wider CIs than those using the multivariable imputed datasets (ESM Tables 5 and 6). In analyses in which those with diagnosed GDM (*n* = 529 Pakistani mothers and 187 white British mothers) were removed, associations of maternal BMI and fasting and post-load glucose with offspring adiposity remained essentially the same as those seen in our main analyses (ESM Table 7). Associations of maternal characteristics with offspring skinfold measurements were broadly similar to those presented in our main analyses with additional adjustment for offspring BMI (Fig. 3b). We found no evidence of differences in the associations of maternal fasting glucose with offspring BMI or SSF or TSF thickness between women with a BMI < 25 kg/m^2^ and those with a BMI ≥ 25 kg/m^2^ in either ethnic group (ESM Table 8).

## Discussion

We have shown that, compared with white British women, Pakistani women have a lower early pregnancy BMI but higher gestational fasting and post-load glucose concentrations and higher prevalence of GDM. At age 4/5 years, their children are taller, have lower BMI and TSF thickness and similar SSF thickness. In both groups of women, greater maternal BMI was associated with higher offspring height, weight, BMI and TSF and SSF thickness, with some evidence of stronger associations in Pakistani compared with white British mother–offspring pairs. There was no evidence that maternal gestational fasting glucose, post-load glucose or a diagnosis of GDM were positively associated with offspring adiposity in either ethnic group in this study. Indeed, point estimates tended to be inverse (i.e. higher maternal glucose associated with lower BMI and skinfold thickness in both groups at age 4/5 years), though CIs were wide and included the null value.

In our previous work with this cohort, we found evidence that Pakistani children have greater relative general adiposity [18]. Our results in this study are broadly similar in that Pakistani children remain lighter and have similar SSF thickness to white British children by age 4/5 years. However, we also see that by this age Pakistani children have lower TSF thickness, which suggests that they may have lower peripheral adiposity (indicated by TSF) but greater central adiposity (indicated by SSF). When we adjusted the ethnic differences in skinfolds for offspring BMI, the difference for SSF thickness increased and became positive, further suggesting greater relative central adiposity. However, this change may be the result of collider bias, given the inverse association of Pakistani ethnicity with BMI and positive association of SSF thickness with BMI, and may be spurious [26].

Our findings are consistent with those of other studies comparing white European and South Asian children; these studies generally found similar or higher skinfold thickness, bioimpedance and fat mass but lower BMI in UK schoolchildren of South Asian origin when compared with those of European origin [13, 28, 29]. We have previously reported that despite smaller size at birth, Pakistani children demonstrate some catch-up in infancy so that by age 2 years they remain lighter but are taller, [25]. Our results reported here for the same children at age 4/5 years suggest that this pattern of ethnic difference in BMI and height continues through early-to-mid childhood. These findings are similar to results from the UK Millennium Cohort Study, which found that Pakistani children were born shorter but were taller at age 5 years than white British children [30] but differ from results of a study of older children aged 9–11 years [13] that compared South Asian children, rather than Pakistani children, with white Europeans, which might explain the different findings.

In previous work, using cord-blood leptin as a marker of total infant fat mass, we found that greater fatness at birth in Pakistani infants compared with white British infants was at least in part mediated by greater gestational glucose in Pakistani women [20]. In the analyses reported here, we found positive associations of maternal BMI with offspring height, weight, BMI and SSF and TSF thickness but we did not identify any robust associations between gestational glucose levels and offspring adiposity at age 4/5 years. The results of previous studies of the association of maternal gestational glycaemia with offspring size/adiposity seem to vary depending on the age at which offspring outcome is assessed. Mendelian randomisation analyses suggest a causal effect of greater maternal gestational glucose on birthweight and ponderal index [2] and matched-within-sibship analyses suggest a causal positive effect by the time offspring are aged 18 years [6]. However, two previous studies of largely white European offspring assessed in infancy found that the positive association of maternal gestational glucose with offspring birth adiposity disappeared within a few months after birth and remained null until at least 24 months of age whereas maternal BMI remained positively associated with offspring adiposity [31, 32]. Similarly, a study of Singapore mother–offspring pairs found that maternal fasting glucose was not associated with adiposity to age 36 months (whereas pre-pregnancy BMI was) [27]. By contrast, a study carried out in the USA found 1 h post (50 g) oral glucose challenge concentrations were positively associated with offspring BMI at age 36 months [33]. Two separate studies of mother and offspring pairs participating in the HAPO (Hyperglycemia and Adverse Pregnancy Outcome) study have reported inconsistent associations between hyperglycaemia and offspring adiposity: participants resident in China showed a positive association with offspring adiposity at age 7 years, especially in girls [21], whereas participants from Northern Ireland (UK) showed no association with offspring adiposity at ages 5–7 years. [8] Taken together, these studies suggest a tendency for the positive effect of maternal gestational hyperglycaemia on birthweight and ponderal index to attenuate in infancy and early childhood at least up to age 4/5 years. Our study, which is considerably larger than previous studies, provides support for this age-related change, though it is possible that the lack of positive associations of GDM and fasting and post-load glucose with offspring adiposity in our study is because those diagnosed with GDM were treated and this treatment attenuates any true positive associations. We do not have information on treatment in the BiB study and so to explore this we repeated analyses after removing any women diagnosed with (and hence treated for) GDM and found results for associations of maternal BMI, fasting and post-load glucose with offspring adiposity remained unchanged. While it is possible that some women who had fasting/post-load glucose values close to the thresholds used for GDM diagnosis were encouraged to modify their diet, they would not have been treated with metformin or insulin and the possible inclusion of such women is unlikely to have attenuated a true positive association to such an extent that point estimates are weakly negative.

While we and others have found positive associations of pre-pregnancy or early pregnancy maternal BMI with offspring subsequent adiposity, including in infancy and early childhood, Mendelian randomisation analyses, negative control studies (comparing maternal and paternal BMI associations with offspring adiposity) and within-sibship analyses, conducted in largely white European populations, suggest that associations of maternal early or pre-pregnancy BMI with subsequent offspring adiposity are unlikely to be due to causal intrauterine effects [6, 34, 35]. Thus, the associations observed here cannot be considered causal. Previous studies have suggested interactions between maternal BMI and gestational glucose measurements in their associations with offspring adiposity [27] and also possible differences between associations in girls and boys [21]. In our larger study, we found no evidence for such interactions.

The key strengths of our study are its large size and the availability of universal OGTT data for all participants. We have measurements of BMI and peripheral and central skinfolds and adjusted for a wide range of potential confounding factors. Up to 23% of offspring had missing outcome data; missing data was more common in white British children than in Pakistani children, reflecting how Bradford’s Pakistani community are more likely to remain in the city and have children in inner-city schools. However, the distributions of outcome measurements were similar in those without missing data and results were similar whether we used the multivariable imputed datasets or the complete case data. Gestational weight gain has also been considered as a possible developmental overnutrition risk factor [1]; however, we do not have data on this in our cohort and so could not explore this here. Our results are from two homogeneous groups and do not necessarily generalise to all South Asians and white Europeans.

In conclusion, at age 4/5 years Pakistani children are taller than white British children but they are lighter and consequently have lower BMI. Assessment of skinfolds suggest that they may have lower levels of peripheral adiposity (lower TSF thickness) but that they may be relatively more centrally adipose (similar SSF thickness). In both ethnic groups, children of mothers with higher BMI were larger and more adipose, with these positive associations tending to be stronger in Pakistani children than in white British children. By contrast, none of the maternal gestational glycaemia measurements were associated with offspring size and adiposity in either ethnic group. As maternal glycaemia is not related to offspring adiposity in either group at 4/5 years it cannot explain ethnic differences in size and adiposity that we, and others, have observed at this age.

## Electronic supplementary material

ESM Tables (PDF 343 kb)

## Abbreviations

BiB: Born in Bradford
GDM: Gestational diabetes
NCMP: National Child Measurement Programme
SSF: Subscapular skinfold thickness
TSF: Triceps skinfold thickness
