# Supplementary material for: Association of maternal exposures with adiposity at age 4/5 years in white British and Pakistani children: findings from the Born in Bradford study
Source: Diabetologia. 2017 Oct 24;61(1):242–52. doi: 10.1007/s00125-017-4457-2 (PMC6046463; doi:10.1007/s00125-017-4457-2)
Supplement: Supplementary file 1 — (PDF 343 kb) [file 125_2017_4457_MOESM1_ESM.pdf]

Electronic Supplementary Material (ESM)
Tables

ESM table 1 comparison of distributions of all characteristics (maternal exposures, offspring outcomes, confounders) from combining imputation datasets and from observed values

Variable	Units or categories	N missing (%)	Observed	Imputed
			Mean (SD) for continuous or % for categorical variables	Mean (SD) for continuous or % for categorical variables
Outcomes				
Maternal BMI	kg/m^2	226 (3.7%)	26.1 (5.8)	26.1 (5.8)
Fasting glucose	mmol/l	224 (3.7%)	4.5 (0.5)	4.5 (0.6)
Post-load glucose	mmol/l	224 (3.7%)	5.7 (1.5)	5.7 (1.6)
Gestational diabetes	No	224 (3.7%)	87.7	87.6
	Yes	224 (3.7%)	12.3	12.4
Maternal exposures and covariables				
Ethnicity (%)	White British	0 (%)	44.8	
	Pakistani	0 (%)	55.2	NA
Age	Years	0 (%)	27.3 (5.6)	NA
Parity (%)	0	189 (3.1%)	38.4	38.2
	1	189 (3.1%)	28.7	28.8
	2	189 (3.1%)	18.0	18.0
	3	189 (3.1%)	9.2	9.2
	4+	189 (3.1%)	5.8	5.8
Education (%)[a]	<5 GCSE	12 (0.2%)	24.1	24.1
	5+ GCSE	12 (0.2%)	33.5	33.4
	A level	12 (0.2%)	14.2	14.2

	Higher than A level	12 (0.2%)	21.7	21.8
	Other	12 (0.2%)	6.6	6.6
Housing tenure (%)[b]	Part/outright owned	10 (0.2%)	64.5	64.5
	Not part/outright owned	10 (0.2%)	35.5	35.5
In receipt of benefits (%)[c]	No	17 (0.3%)	56.0	56.0
	Yes	17 (0.3%)	44.0	44.0
Smoking (%)[d]	No	12 (0.2%)	82.1	82.0
	Yes	12 (0.2%)	17.9	18.0
Offspring exposures and covariables				
Height	Cm	918 (15.1%)	108.4 (5.0)	108.4 (5.4)
Weight	Kg	878 (14.5%)	19.0 (3.0)	19.0 (3.2)
BMI	kg/m^2	918 (15.1%)	16.1 (1.7)	16.1 (1.8)
Subscapular skinfold	Mm	1442 (23.8%)	6.3 (2.2)	6.3 (2.9)
Triceps skinfold	Mm	1390 (22.9%)	10.1 (3.3)	10.1 (3.8)
Age at measurement	Years	1384 (22.8%)	5.1 (0.3)	5.1 (0.3)
Gender (%)	Male	0 (0%)	50.5	NA
	Female		49.5	

SD: standard deviation; NA: not applicable (for these there is no missing data)
[a]We equivalised the mother's highest educational qualifications (based on the qualification received and the country obtained) to UK standard attainments using 5 mutually exclusive categories as previously reported.[18] [b]Housing tenure was defined as whether the woman lived in a household where the home was either part-owned (i.e. mortgaged) or owned outright, or not (i.e. rented). [c]Receipt of means tested benefits was defined as anyone in the household receiving state means tested benefits. [d]Smoking was categorised for this study as (1) never smoking during pregnancy or (2) smoking at any time during pregnancy.

ESM table 2 Child age and sex adjusted associations of maternal pregnancy exposures with offspring height, weight and adiposity (model 1)

Measure	Maternal BMI *Difference in means per 1 kg/m²*			Fasting glucose *Difference in means per 1mmol/l*			Post-load glucose *Difference in means per 1mmol/l*			Gestational diabetes		
	White British N=2717	*Pakistani N=3343*	*p value for ethnic interaction[a]*	*White British N=2717*	*Pakistani N=3343*	*p value for ethnic interaction[a]*	*White British N=2717*	*Pakistani N=3343*	*p value for ethnic interaction[a]*	*White British N=2717*	*Pakistani N=3343*	*p value for ethnic interaction[a]*
Height (cm)	0.064 (0.033, 0.096)	0.058 (0.027, 0.090)	0.801	0.189 (-0.265, 0.643)	0.062 (-0.259, 0.385)	0.618	-0.120 (-0.274, 0.033)	-0.081 (-0.194, 0.033)	0.720	-0.284 (-1.056, 0.489)	0.166 (-0.343, 0.675)	0.375
Weight (kg)	0.095 (0.077, 0.113)	0.131 (0.109, 0.152)	0.014	-0.039 (-0.298, 0.219)	-0.149 (-0.359, 0.062)	0.823	-0.083 (-0.168, 0.001)	-0.060 (-0.131, 0.010)	0.323	-0.086 (-0.519, 0.348	-0.164 (-0.495, 0.167)	0.728
BMI (kg/m^2)	0.060 (0.051, 0.070)	0.089 (0.077, 0.102)	0.001	-0.088 (-0.237, 0.061)	-0.145 (-0.259, 0.030)	0.505	-0.036 (-0.081, 0.009)	-0.026 (-0.065, 0.012)	0.241	0.017 (-0.223, 0.258)	-0.186 (-0.371, 0.001)	0.733
SSF (mm)	0.062 (0.048, 0.075)	0.079 (0.064, 0.096)	0.094	0.062 (0.047, 0.076)	-0.115 (-0.271, 0.041)	0.833	0.020 (-0.043, 0.084)	0.001 (-0.055, 0.059)	0.228	0.064 (-0.276, 0.403)	-0.189 (-0.437, 0.057)	0.487
TSF (mm)	0.064 (0.044, 0.084)	0.081 (0.059, 0.104)	0.242	0.068 (0.046, 0.089)	-0.068 (-0.300, 0.163)	0.341	0.067 (0.046, 0.087)	0.006 (-0.070, 0.083)	0.192	-0.253 (-0.783, 0.278)	-0.118 (-0.474, 0.238)	0.482

All results are adjusted for offspring sex, and age at measurement (Model 1). Analyses were based on the multiple imputed datasets.
a Testing the null hypothesis that there is no difference in associations of maternal characteristics with offspring outcomes between White British and Pakistani

ESM table 3a: Adjusted associations of maternal pregnancy exposures with offspring height, weight and adiposity in White British participants stratified by gender (model 2)

Measure	Maternal BMI *Difference in means per 1 kg/m^2*			Fasting glucose *Difference in means per 1mmol/l*			Post-load glucose *Difference in means per 1mmol/l*			Gestational diabetes		
	Boys N=1381	*Girls N=1336*	*p value for gender interaction*[a]	*Boys N=1381*	*Girls N=1336*	*p value for gender interaction*[a]	*Boys N=1381*	*Girls N=1336*	*p value for gender interaction*[a]	*Boys N=1381*	*Girls N=1336*	*p value for gender interaction*[a]
Height (cm)	0.050 (0.009, 0.091)	0.074 (0.029, 0.120)	0.553	-0.160 (-0.747. 0.427)	0.055 (-0.576, 0.687)	0.553	-0.145 (-0.347, 0.057)	0.053 (-0.154, 0.259)	0.212	-0.735 (-1.675, 0.204)	-0.145 (-1.233, 0.943)	0.408
Weight (kg)	0.085 (0.059, 0.110)	0.115 (0.087, 0.143)	0.254	-0.083 (-0.433, 0.267)	-0.036 (-0.440, 0.367)	0.509	-0.172 (-0.288, -0.056)	-0.004 (-0.133, 0.125)	0.058	-0.331 (-0.891, 0.230)	0.144 (-0.515, 0.803)	0.178
BMI (kg/m^2)	0.056 (0.042, 0.070)	0.073 (0.057, 0.089)	0.199	-0.067 (-0.265, 0.132)	-0.036 (-0.267, 0.195)	0.547	-0.053 (-0.115, 0.008)	0.021 (-0.052, 0.094)	0.075	-0.070 (-0.386, 0.247)	0.197 (-0.171,	0.171
SSF (mm)	0.055 (0.037, 0.072)	0.077 (0.055, 0.099)	0.193	0.059 (-0.216, 0.334)	0.010 (-0.323, 0.343)	0.832	-0.044 (-0.121, 0.034)	0.009 (-0.095, 0.114)	0.315	0.060 (-0.362, 0.482)	0.154 (-0.385, 0.693)	0.580
TSF (mm)	0.075 (0.046, 0.104)	0.069 (0.037, 0.100)	0.855	-0.056 (-0.502, 0.391)	-0.248 (-0.760, 0.263)	0.683	-0.099 (-0.227, 0.029)	0.029 (-0.121, 0.180)	0.173	-0.286 (-0.983, 0.411)	-0.070 (-0.873, 0.732)	0.690

All results are adjusted for offspring sex, and age at measurement, maternal age, parity, BMI (for maternal fasting and post-load glucose and GDM), maternal height (when the outcome was offspring height), education, housing tenure, whether anyone in the household was in receipt of means tested benefits, and smoking (Model 2). Analyses were based on the multiple imputed datasets.

a Testing the null hypothesis that there is no difference in associations of maternal characteristics with offspring outcomes between boys and girls

ESM table 3b: Adjusted associations of maternal pregnancy exposures with offspring height, weight and adiposity in Pakistani participants stratified by gender (model 2)

Measure	Maternal BMI *Difference in means per 1 kg/m^2*			Fasting glucose *Difference in means per 1mmol/l*			Post-load glucose *Difference in means per 1mmol/l*			Gestational diabetes		
	Boys N=1681	*Girls N=1662*	*p value for gender interaction*[a]	*Boys N=1681*	*Girls N=1662*	*p value for gender interaction*[a]	*Boys N=1681*	*Girls N=1662*	*p value for gender interaction*[a]	*Boys N=1681*	*Girls N=1662*	*p value for gender interaction*[a]
Height (cm)	0.079 (0.033, 0.125)	0.090 (0.045, 0.136)	0.377	0.117 (-0.328. 0.562)	0.013 (-0.391, 0.416)	0.908	-0.004 (-0.152, 0.160)	-0.009 (-0.168, 0.150)	0.614	-0.603 (-0.080, 1.286)	0.113 (-0.554, 0.780)	0.580
Weight (kg)	0.148 (0.116, 0.180)	0.152 (0.121, 0.184)	0.246	-0.029 (-0.338, 0.279)	-0.241 (-0.512, 0.029)	0.704	-0.039 (-0.140, -0.062)	-0.060 (-0.161, 0.041)	0.650	0.111 (-0.370, 0.592)	-0.305 (-0.742, 0.132)	0.595
BMI (kg/m^2)	0.097 (0.079, 0.115)	0.101 (0.083, 0.119)	0.268	-0.071 (-0.239, 0.098)	-0.200 (-0.350, -0.050)	0.588	-0.014 (-0.069, 0.042)	-0.024 (-0.079, 0.030)	0.772	-0.082 (-0.360, 0.196)	-0.227 (-0.468, 0.014)	0.803
SSF (mm)	0.074 (0.050, 0.097)	0.113 (0.087, 0.139)	0.030	-0.056 (-0.283, 0.171)	-0.143 (-0.366, 0.080)	0.942	-0.021 (-0.099, 0.056)	0.049 (-0.037, 0.135)	0.139	-0.038 (-0.364, 0.288)	-0.229 (-0.593, 0.134)	0.757
TSF (mm) *Boys*	0.096 (0.064, 0.128)	0.105 (0.067, 0.141)	0.699	-0.032 (-0.379, 0.315)	-0.052 (-0.368, 0.264)	0.948	0.010 (-0.099, 0.118)	0.053 (-0.063, 0.169)	0.580	-0.353 (-0.353, 0.663)	-0.200 (-0.710, 0.310)	0.417

All results are adjusted for offspring sex, and age at measurement, maternal age, parity, BMI (for maternal fasting and post-load glucose and GDM), maternal height (when the outcome was offspring height), education, housing tenure, whether anyone in the household was in receipt of means tested benefits, and smoking (Model 2). Analyses were based on the multiple imputed datasets.

a Testing the null hypothesis that there is no difference in associations of maternal characteristics with offspring outcomes between boys and girls

ESM table 4
Adjusted associations between maternal pregnancy exposures and child adiposity with both ethnic groups combined using multivariable imputed datasets (n=6060)

Measure	Maternal BMI *Difference in means per 1 kg/m²*	Fasting glucose *Difference in means per 1mmol/l*	Post-load glucose *Difference in means per 1mmol/l*	Gestational diabetes
Height (cm)	0.073 (0.051, 0.095)	0.035 (-0.212, 0.282)	-0.025 (-0.112, 0.062)	0.102 (-0.319, 0.524)
Weight (kg)	0.124 (0.109, 0.139)	-0.095 (-0.264, 0.075)	-0.065 (-0.119, -0.009)	-0.096 (-0.373, 0.181)
BMI (kg/m²)	0.081 (0.072, 0.089)	-0.098 (-0.193, -0.003)	-0.016 (-0.046, -0.013)	-0.084 (-0.234, 0.065)
SSF (mm)	0.078 (0.067, 0.089)	-0.055 (-0.177, 0.061)	-0.005 (-0.038, 0.047)	-0.063 (-0.273, 0.136)
TSF (mm)	0.085 (0.069, 0.101)	-0.056 (-0.251, -0.139)	-0.007 (-0.056, -0.070)	-0.078 (-0.375, -0.219)

All results are adjusted for offspring sex, and age at measurement, maternal age, parity, BMI (for maternal fasting and post-load glucose and GDM), maternal height (when the outcome was offspring height), education, housing tenure, whether anyone in the household was in receipt of means tested benefits, and smoking (Model 2). Analyses were based on the multiple imputed datasets.

ESM table 5

Adjusted associations based on complete case analyses presented as mean differences for both groups stratified by ethnicity with interaction p-values

Measure	Maternal BMI *Difference in means per 1 kg/m^2*			Fasting glucose *Difference in means per 1mmol/l*			Post-load glucose *Difference in means per 1mmol/l*			Gestational diabetes		
	White British N=1725	*Pakistani N=1941*	*p value for ethnic interaction* [a]	*White British N=1725*	*Pakistani N=1941*	*p value for ethnic interaction* [a]	*White British N=1725*	*Pakistani N=1941*	*p value for ethnic interaction* [a]	*White British N=1725*	*Pakistani N=1941*	*p value for ethnic interaction* [a]
Height (cm)	0.063 (0.028, 0.098)	0.062 (.022, 0.101)	0.997	-0.369 (-0.871, 0.132)	0.218 (-0.138, 0.575)	0.102	-0.166 (-0.326, -0.006)	-0.003 (-0.131, 0.124)	0.162	-0.800 (-1.62, 0.020)	0.597 (0.039, 1.154)	0.009
Weight (kg)	0.094 (0.073, 0.115)	0.125 (0.099, 0.152)	0.086	-0.184 (-0.487, 0.119)	-0.008 (-0.243, 0.228)	0.272	-0.139 (-0.235, -0.042)	-0.055 (-0.139, 0.028)	0.211	-0.176 (-0.673, 0.319)	-0.027 (-0.396, 0.340)	0.503
BMI (kg/m^2)	0.060 (0.049, 0.072)	0.088 (0.073, 0.103)	0.006	-0.062 (-0.228, 0.104)	-0.071 (-0.203, 0.061)	0.516	-0.026 (-0.079, 0.027)	-0.022 (-0.069, 0.025)	0.631	0.081 (-0.191, 0.353)	-0.166 (-0.372, 0.041)	0.405
SSF (mm)	0.055 (0.039, 0.070)	0.067 (0.046, 0.087)	0.396	0.026 (-0.193, 0.245)	-0.093 (-0.277, 0.089)	0.757	-0.023 (-0.092, 0.047)	0.006 (-0.059, 0.071)	0.381	0.137 (-0.222, 0.496)	-0.164 (-0.451, 0.122)	0.341
TSF (mm)	0.057 (0.033, 0.082)	0.070 (0.042, 0.098)	0.480	-0.237 (-0.588, 0.115)	0.005 (-0.248, 0.257)	0.176	-0.066 (-0.178, 0.046)	0.004 (-0.086, 0.094)	0.248	-0.362 (-0.938, 0.213)	-0.109 (-0.504, 0.286)	0.347

All results are adjusted for offspring sex, and age at measurement, maternal age, parity, BMI (for maternal fasting and post-load glucose and GDM), maternal height (when the outcome was offspring height), education, housing tenure, whether anyone in the household was in receipt of means tested benefits, and smoking (Model 2).

a Testing the null hypothesis that there is no difference in associations of maternal characteristics with offspring outcomes between White British and Pakistani

ESM table 6
Adjusted associations based on complete case analyses presented as mean differences for both groups combined (n=3666)

Measure	Maternal BMI *Difference in means per 1 kg/m^2*	Fasting glucose *Difference in means per 1mmol/l*	Post-load glucose *Difference in means per 1mmol/l*	Gestational diabetes
Height (cm)	0.063 (0.036, 0.089)	0.032 (-0.255, 0.321)	-0.065 (-0.165, .033)	0.167 (-0.289, 0.624)
Weight (kg)	0.108 (0.092, 0.125)	-0.063 (-0.247, 0.120)	-0.089 (-0.151, -0.026)	-0.065 (-0.357, 0.225)
BMI (kg/m^2)	0.073 (0.064, 0.082)	-0.066 (-0.168, 0.036)	-0.024 (-0.059, 0.011)	-0.079 (-0.241, 0.082)
SSF (mm)	0.060 (0.047, 0.073)	-0.057 (-0.196, 0.081)	-0.004 (-0.051, 0.043)	-0.069 (-0.289, 0.149)
TSF (mm)	0.062 (0.044, 0.080)	-0.072 (-0.276, 0.131)	-0.022 (-0.092, 0.048)	-0.179 (-0.501, 0.144)

All results are adjusted for offspring sex, and age at measurement, maternal age, parity, BMI (for maternal fasting and post-load glucose and GDM), maternal height (when the outcome was offspring height), education, housing tenure, whether anyone in the household was in receipt of means tested benefits, and smoking (Model 2).

ESM table 7

Adjusted associations between maternal pregnancy exposures and child adiposity by ethnicity using multivariable imputed datasets excluding women with GDM

Measure	Maternal BMI *Difference in means per 1 kg/m²*			Fasting glucose *Difference in means per 1mmol/l*			Post-load glucose *Difference in means per 1mmol/l*		
	White British N=2530	*Pakistani N=2814*	*p value for ethnic interaction*[a]	*White British N=2530*	*Pakistani N=2814*	*p value for ethnic interaction*[a]	*White British N=2530*	*Pakistani N=2814*	*p value for ethnic interaction*[a]
Height (cm)	0.066 (0.034, 0.099)	0.086 (0.048, 0.123)	0.568	0.248 (-0.354, 0.851)	0.037 (-0.546, 0.620)	0.648	0.039 (-0.129, 0.208)	-0.056 (-0.225, 0.112)	0.388
Weight (kg)	0.101 (0.081, 0.121)	0.161 (0.135, 0.186)	0.001	0.063 (-0.299, 0.425)	-0.004 (-0.387, 0.379)	0.827	-0.075 (-0.171, 0.021)	-0.078 (-0.191, 0.035)	0.886
BMI (kg/m^2)	0.065 (0.053, 0.076)	0.108 (0.093, 0.122)	0.000	-0.024 (-0.223, 0.176)	-0.033 (-0.249, 0.183)	0.460	-0.013 (-0.066, 0.039)	-0.014 (-0.078, 0.049)	0.682
SSF (mm)	0.065 (0.050, 0.079)	0.101 (0.081, 0.121)	0.010	0.032 (-0.226, 0.289)	-0.108 (-0.401, 0.184)	0.921	-0.016 (-0.092, 0.060)	0.003 (-0.087, 0.093)	0.520
TSF (mm)	0.075 (0.053, 0.098)	0.109 (0.082, 0.136)	0.087	-0.001 (-0.425, 0.424)	0.049 (-0.388, 0.486)	0.635	-0.008 (-0.118, 0.101)	0.042 (-0.077, 0.160)	0.517

All results are adjusted for offspring sex, and age at measurement, maternal age, parity, BMI (for maternal fasting and post-load glucose and GDM), maternal height (when the outcome was offspring height), education, housing tenure, whether anyone in the household was in receipt of means tested benefits, and smoking (Model 2). Analyses were based on the multiple imputed datasets.

a Testing the null hypothesis that there is no difference in associations of maternal characteristics with offspring outcomes between White British and Pakistani

ESM table 8 Adjusted associations between maternal fasting glucose and child adiposity by ethnicity using multivariable imputed datasets with results presented separately for women with a BMI≥25 and those with a BMI<25

Measure	Fasting glucose<br>*Difference in means per 1mmol/l*					
	White British<br>N=2717			*Pakistani<br>N=3343*		
	BMI<br><25<br>N = 1198	*BMI<br>≥25<br>N = 1519*	*p-value for interaction[a]*	*BMI<br><25<br>N = 1678*	*BMI<br>≥25<br>N = 1665*	*p-value for interaction[a]*
BMI (kg/m^2)	-0.036<br>(-0.308, 0.237)	-0.059<br>(-0.255, 0.138)	0.657	-0.005<br>(-0.216, 0.206)	-0.180<br>(-0.318, -0.042)	0.934
SSF (mm)	0.164<br>(-0.157, 0.485)	-0.028<br>(-0.295, 0.239)	0.539	0.014<br>(-0.278, 0.307)	-0.139<br>(-0.325 0.048)	0.281
TSF (mm)	0.073<br>(-0.484, 0.629)	-0.242<br>(-0.667, 0.182)	0.348	0.197<br>(-0.219, 0.614)	-0.107<br>(-0.379, 0.164)	0.167

All results are adjusted for offspring sex, and age at measurement, maternal age, parity, BMI (for maternal fasting and post-load glucose and GDM), maternal height (when the outcome was offspring height), education, housing tenure, whether anyone in the household was in receipt of means tested benefits, and smoking (Model 2). Analyses were based on the multiple imputed datasets.
a Testing the null hypothesis that there is no difference in associations of BMI with offspring outcomes between the 2 BMI groups (<25; >25)
